# Epidermal growth factor receptor–targeted near-infrared photoimmunotherapy for epidermal growth factor receptor–expressing advanced solid tumors: a scoping review of barriers and enablers to clinical translation

**DOI:** 10.1093/abt/tbag026

**Published:** 2026-06-05

**Authors:** Emmanuel O Oisakede, Olawunmi O Oyedeji, Odunola F Atitebi, Ijomah Ugbomah, David B Olawade

**Affiliations:** Department of Clinical Oncology, Leeds Teaching Hospitals NHS Trust, Leeds, LS9 7TF, United Kingdom; Department of Health Research, University of Leeds, Leeds, LS2 9JT, United Kingdom; Department of Research and Innovation, The Christie NHS Foundation Trust, Manchester, M20 4BX, United Kingdom; Can-Survive UK, Manchester, M15 5DD, United Kingdom; Department of Transformation and Quality Improvement, University Hospitals of Northamptonshire, Kettering, NN16 8UZ, United Kingdom; Department of Research and Innovation, Medway NHS Foundation Trust, Gillingham ME75NY, United Kingdom; Department of Public Health, York St John University, London, E14 2BA, United Kingdom; Department of Allied and Public Health, School of Health, Sport and Bioscience, University of East London, London, E16 2RD, United Kingdom

**Keywords:** near-infrared photoimmunotherapy, EGFR-targeted therapy, head and neck squamous cell carcinoma, drug-device combination, clinical translation, photodynamic therapy, Cetuximab sarotalocan, IR700

## Abstract

**Background:**

Epidermal growth factor receptor (EGFR)-targeted near-infrared photoimmunotherapy (NIR-PIT) is an emerging drug-device modality that enables spatially selective tumor cell killing through antibody-photoabsorber conjugation and localized light activation. Although clinical translation has advanced in selected settings, particularly head and neck squamous cell carcinoma (HNSCC), barriers and enablers influencing broader implementation across solid tumors have not been systematically mapped. This review focuses specifically on the IR700 (IRDye700DX)-based conjugate cetuximab sarotalocan (RM-1929/ASP-1929), which is the only NIR-PIT construct to have advanced into clinical use, while acknowledging the broader and rapidly expanding photoimmunotherapy landscape.

**Objective:**

To synthesize and characterize the biological, technical, clinical, and system-level factors that facilitate or hinder the clinical translation of EGFR-targeted NIR-PIT across EGFR-expressing advanced solid tumors.

**Methods:**

A scoping review was conducted in accordance with PRISMA-ScR guidelines. Comprehensive searches of PubMed, Embase, Scopus, ClinicalTrials.gov, and the Cochrane Library identified 28 eligible studies, including preclinical investigations, early-phase clinical trials, observational studies, and case reports or series. Data were charted and synthesized thematically, with study quality appraised to inform interpretation.

**Results:**

Preclinical studies consistently demonstrated light-dependent, EGFR-selective cytotoxicity and identified non-linear dose–response relationships that support standardized light dosing strategies. Clinical translation has been most successful in recurrent or unresectable HNSCC, facilitated by tumor accessibility, multidisciplinary workflows, and regulatory approval in Japan. Technical enablers included endoscopic and navigation-assisted light delivery, real-time fluorescence imaging, and the feasibility of repeat treatment without cumulative toxicity. Key barriers included limited light penetration, anatomical constraints, reliance on specialized equipment, and restricted applicability to deep-seated or diffuse disease. Although most adverse events were localized and manageable, rare but serious complications reported across multiple case studies represent an important translational challenge. Evidence outside HNSCC remains sparse and is largely confined to preclinical models.

**Conclusions:**

EGFR-targeted NIR-PIT has achieved meaningful clinical translation within a narrow but well-defined therapeutic niche, supported by strong mechanistic rationale and growing real-world experience. Broader adoption is constrained by biological, technical, regulatory and system-level barriers, as well as gaps in comparative effectiveness, long-term outcomes, and health-economic evidence. Addressing these challenges through targeted clinical trials, improved patient selection, and implementation-focused research will be essential to advance NIR-PIT toward wider clinical integration.

**Statement of Significance**

This scoping review is the first to systematically map biological, technical, clinical, and regulatory barriers and enablers shaping the translation of EGFR-targeted near-infrared photoimmunotherapy. By reframing efficacy evidence through an implementation lens, it provides a practical framework to guide future clinical adoption and translational research.

## Introduction

Advanced solid tumors remain a leading cause of cancer morbidity and mortality, and many patients eventually develop disease that is refractory to standard surgery, radiotherapy, chemotherapy, and immunotherapy. One strategy to expand therapeutic options is to exploit cell-surface targets that are overexpressed across multiple solid tumour types. Epidermal growth factor receptor (EGFR) is one such target, with clinically established targeting via monoclonal antibodies in several cancers and broad relevance across solid tumors [1]. In head and neck squamous cell carcinoma (HNSCC), EGFR is expressed in about 90% of cases [2].

Near-infrared photoimmunotherapy (NIR-PIT) is a drug-device therapeutic modality designed to achieve highly localized tumour cell killing through a targeted antibody-photo absorber conjugate followed by near-infrared light activation at the tumour site [3, 4]. Mechanistically, NIR-PIT has been described as inducing rapid, selective tumour cell death and can trigger immunogenic cell death and host anti-tumour immune responses, supporting interest in combination strategies with immune checkpoint inhibitors [5].

The most clinically advanced EGFR-targeted NIR-PIT agent is RM-1929/ASP-1929 (cetuximab sarotalocan), which consists of cetuximab (anti-EGFR) conjugated to the photoabsorber dye IRDye700DX (IR700) and is activated using a dedicated light delivery system [6]. Combination with other immunotherapy have shown similar feasibility and tolerance profile [7, 8]. Clinical development to date has been anchored in recurrent/metastatic or locoregionally recurrent HNSCC, including phase I and phase 1/2a studies reporting feasibility, safety, and signals of anti-tumor activity in heavily pre-treated populations [6, 9]. In Japan, ASP-1929 photoimmunotherapy received marketing approval for specific head and neck cancer indications under an accelerated/conditional framework, reflecting both maturity of the platform and the regulatory complexity of a combined drug-device intervention [10]. At the same time, expansion beyond HNSCC to other EGFR-expressing advanced solid tumors raises translational questions spanning (i) biological considerations (target expression heterogeneity, tumor microenvironment), (ii) technical constraints (light delivery, dosimetry, accessibility of lesions), (iii) clinical workflow and infrastructure (interdisciplinary delivery in operating rooms/endoscopy suites), (iv) safety and patient selection, and (v) regulatory, reimbursement, and implementation pathways for a drug-device combination. Emerging clinical studies also explore combination approaches (e.g. with pembrolizumab) [7], further emphasizing the need to understand translational enablers and barriers across the evolving evidence base.

The aim of this review is to map and synthesize reported barriers and enablers to clinical translation of EGFR-targeted NIR-PIT across EGFR-expressing advanced solid tumors, drawing on evidence from clinical trials, case reports, observational studies, and preclinical studies.

### Objectives

- Identify and categorize barriers to translation reported or inferable from primary studies
- Identify and categorize enablers
- Summarize reported outcomes used to support translation (feasibility, safety, tumor response metrics, patient-relevant outcomes, workflow endpoints, and translational immunology endpoints where available).
- Identify knowledge gaps and research priorities needed to advance clinical translation across tumor sites (including where evidence is limited to preclinical studies).

## Methods

This scoping review was conducted and reported in accordance with the Preferred Reporting Items for Systematic Reviews and Meta-Analyses extension for Scoping Reviews (PRISMA-ScR) [11].

### Literature search

A comprehensive literature search was undertaken to identify relevant studies on EGFR-targeted NIR-PIT near-infrared photoimmunotherapy (RM-1929/ASP-1929) and factors influencing its clinical translation. The following databases and trial registry were searched: PubMed, Scopus, Embase, ClinicalTrials.gov, and The Cochrane Library. The literature search spanned published studies from the period between January 2015 to January 2026. Search terms were developed using controlled vocabulary (where applicable) and free-text keywords related to near-infrared photoimmunotherapy and EGFR-targeted agents (e.g. “near-infrared photoimmunotherapy,” “NIR-PIT,” “photoimmunotherapy,” “RM-1929,” “ASP-1929,” “cetuximab sarotalocan,” “IRDye700DX/IR700”), combined with terms related to translation and implementation where relevant. See (appendix S1 in the supplemental 1 file) for the full electronic search strategy attached as supplementary file. Following reviewer feedback, the search was updated to incorporate broader photoimmunotherapy-related terminology, specifically including “photodynamic therapy” (PDT) as a related construct, as well as additional terms for emerging photoimmunotherapy platforms (e.g. “virus-like drug conjugate,” “VDC,” “belzupacap sarotalocan,” “NIRa Biosciences,” “Daiichi Sankyo photoimmunotherapy”) and relevant patent, poster, and press release literature from Rakuten Medical, Aura Biosciences, NIRa Biosciences, and Daiichi Sankyo to capture clinical developments not yet indexed in peer-reviewed databases. Although, studies obtained from the search update did not meet the inclusion criteria, but were relevant to enrich the discussion in this study.

## Eligibility criteria

### Inclusion criteria

Studies were included if they:

- Were primary research (published articles or trial registry records with primary study details).
- Investigated EGFR-targeted near-infrared photoimmunotherapy, specifically RM-1929/ASP-1929 (cetuximab sarotalocan), or directly evaluated its use in combination with an NIR light delivery system.
- Examined solid tumors in any setting, including:
  Human studies (any stage of clinical development), and/or
  Preclinical studies (animal models) relevant to translation.
- Were any of the following study designs:
  Observational studies: cohort, case–control, cross-sectional, and case reports/case series
  Experimental studies: including randomized controlled trials (RCTs) and animal studies
  Quasi-experimental studies
- Reported data relevant to the review aims, including intervention delivery characteristics, feasibility, safety, efficacy signals, workflow considerations, device/light delivery aspects, and/or information that could inform barriers or enablers to clinical translation.

### Exclusion criteria

Studies were excluded if they:

- Were not primary research, such as narrative reviews, systematic reviews, scoping reviews, editorials, commentaries, letters without primary data, protocols without results (unless used only to map ongoing studies), conference abstracts lacking sufficient methodological detail, or opinion pieces.
- Did not involve EGFR-targeted NIR-PIT using RM-1929/ASP-1929 (cetuximab sarotalocan) (e.g. studies of other photoimmunotherapy platforms without RM-1929/ASP-1929, or photodynamic therapy not classed as NIR-PIT).
- Focused on non-solid tumors, in vitro-only experiments without translational relevance, or purely technical optics/device bench studies with no link to RM-1929/ASP-1929 use in tumor models or patients.
- Were duplicates or secondary publications of the same dataset (in such cases, the most complete or most recent report was retained).

**Figure 1:**
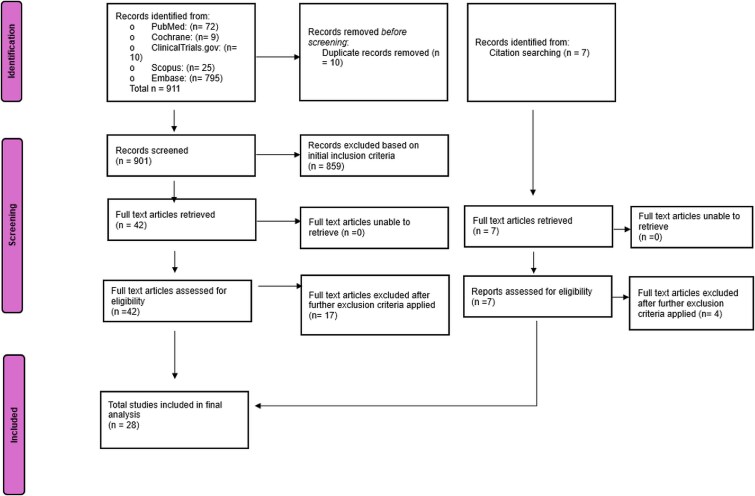
PRISMA-ScR flowchart.

Following duplicate removal using the Covidence software, all studies (firstly title, abstract, full text) were screened against the identified eligibility criteria. See PRISMA flowchart illustrating the literature search and selection process (Fig. 1).

**Figure 2:**
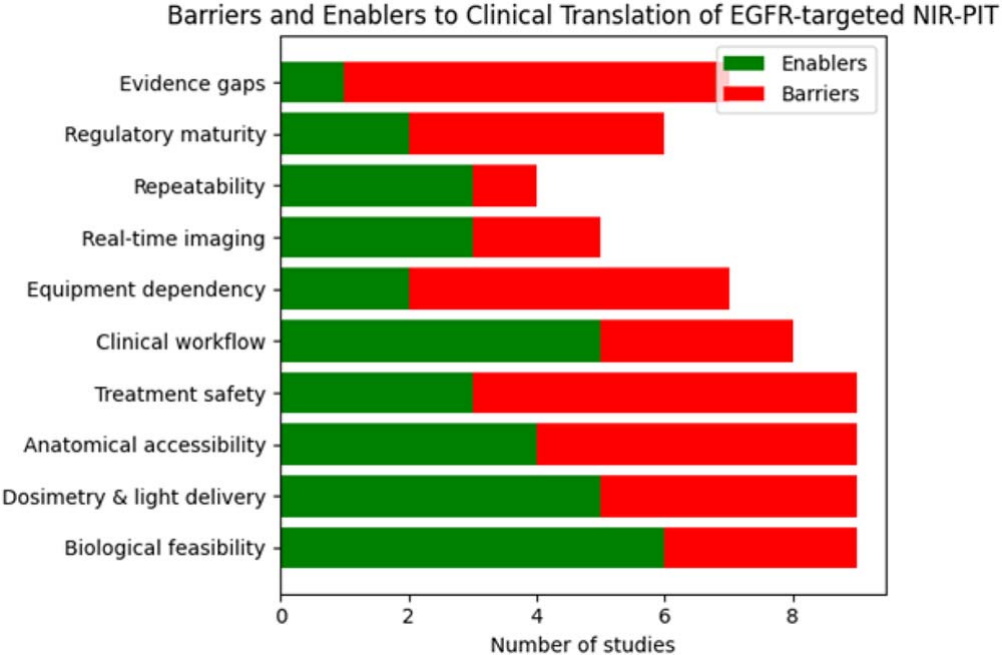
Barriers and enablers to clinical translation of EGFR-targeted NIR-PIT.

## Data extraction

Data were extracted using a standardized extraction sheet developed for this review. Extracted information included study characteristics (design, setting, population/model, tumor type), intervention and light-delivery parameters, outcomes, and reported (or inferable) barriers and enablers relevant to clinical translation. The team piloted the extraction tool on the set of included articles to confirm it captured information relevant to the scoping review question and then refined the form accordingly [12]. The finalized extraction was subsequently completed using the Covidence tool.

To ensure consistency and resolve uncertainty, studies with unclear eligibility, ambiguous reporting, or extraction concerns were discussed among multiple authors (EOO and OOO), and discrepancies were resolved through consensus.

## Data synthesis and analysis

A thematic synthesis was performed to provide a robust overview of the data from the included research studies. This approach was deemed suitable due to the heterogeneity of study designs, encompassing preclinical animal studies, randomized and non-randomized clinical trials, observational studies, and case reports/series, and the diversity of intervention regimens, including Cetuximab-Sarotalocan (RM-1929/ASP-1929) and combination protocols such as ASP-1929 with Pembrolizumab. Variations in tumor types, light-delivery systems, and outcome reporting further precluded statistical pooling or meta-analysis.

Data were synthesized into themes. Quantitative findings such as safety signals, feasibility metrics, and response rates were summarized using narrative integration, while qualitative and mechanistic insights (e.g. workflow adaptations, regulatory context, immune activation) were categorized thematically. The synthesis focused on identifying barriers and enablers to clinical translation, drawing on both explicit statements and inferable factors across biological, technical, clinical workflow, regulatory, and implementation domains.

To ensure rigor, data charting was guided by scoping review methodology standards. Findings from each study were independently verified and cross-compared for conceptual convergence and divergence. The extracted data were iteratively mapped to an analytical framework structured around translational determinants, enabling an integrated interpretation across preclinical and clinical evidence streams.

Where applicable, emerging cross-cutting themes such as immune modulation, dosimetry standardization, treatment accessibility, and real-time fluorescence monitoring were highlighted as translational enablers, while issues such as light penetration depth, intertumoral EGFR heterogeneity, adverse event management, and infrastructure limitations were identified as recurrent barriers.

## Quality assessment

Quality appraisal was conducted using validated, study-design-specific tools to ensure methodological rigour across the diverse body of included evidence. Among the included works, seven were clinical trials or observational human studies, ten were single-patient case reports, four were retrospective or quality-of-life studies, and seven were preclinical experimental studies exploring mechanistic or optimisation aspects of NIR-PIT.

For non-randomized clinical trials and early-phase interventional studies, the Cochrane ROBINS-I Checklist was applied [13]. Observational human studies, including QoL, prospective and retrospective cohort designs, were evaluated using the CASP Cohort Study Checklist [14]. Single-patient case reports were assessed using the Joanna Briggs Institute’s (JBI) critical appraisal checklist for case reports, while case series were appraised using the JBI case series checklist [15]. Preclinical animal studies were evaluated using the SYRCLE Risk of Bias Tool [16], which is specifically designed to assess methodological quality in animal experimentation.

The report of all quality assessments classified by each study design are summarized in Appendix S2–S6

## Results

### Summary of included studies

A total of 28 studies published between 2015 and 2026 were included in this scoping review, consisting of a spectrum of preclinical, clinical, and translational investigations of EGFR-targeted NIR-PIT. Most of the studies originated from Japan (n = 24), reflecting the country’s leading role in clinical development and implementation of cetuximab sarotalocan sodium (RM-1929/ASP-1929). Additional studies were conducted in the United States (n = 4). See Table 1 for study distribution across countries and characteristics.

**Table 1:** Study characteristics.

Author	Country	Treatment	Study Design	Tumor Type	Sample Size	Light Parameters	Key Outcomes
Tahara et al. [6]	Japan	RM-1929 (Cetuximab-IR700)	Phase I Clinical Trial	Recurrent HNSCC	n = 3	50 J/cm^2^ superficial; 100 J/cm diffuser	Manageable safety; 2 partial responses observed
Cognetti et al. [7]	USA	cetuximab sarotalocan plus Pembrolizumab	Phase Ib/II Clinical Trial	Recurrent/Metastatic HNSCC	n = 18	690 nm	Safety and preliminary efficacy of PIT combined with PD-1 blockade
Kadota et al. [8]	Japan	Cetuximab-Sarotalocan plus nivolumab	Phase Ib clinical trial	EGFR-expressing gastric cancer	n = 21	690 nm with escalation 50 > 75 > 100 J/cm2; endoscopic illumination	Safety and feasibility of PIT combined with PD-1 inhibition
Cognetti et al. [9]	USA	RM-1929 (Cetuximab-IR700)	Phase I/IIa Clinical Trial	Recurrent/Metastatic HNSCC	n = 39	50 J/cm^2^ superficial; 100 J/cm diffuser	Feasibility, safety, 43% ORR
Okamoto et al. [17]	Japan	Cetuximab-Sarotalocan (RM-1929)	Case report	Recurrent laryngeal carcinoma involving cervical lymph node lesion.	n = 1	50 J/cm^2^ superficial; 100 J/cm diffuser	Complete local response; functional preservation
Okamoto et al. [18]	Japan	Cetuximab-Sarotalocan (RM-1929)	QoL Study	Recurrent HNSCC	n = 9	50 J/cm^2^	Improved QoL; transient pain & swelling
Okamoto et al. [19]	Japan	CRM-1929 with navigation-assisted interstitial light delivery	Case report	Recurrent HNSCC (lateral pterygoid)	n = 1	50–100 J/cm^2^ (interstitial)	Navigation-guided PIT feasibility; precise targeting
Nishikawa et al. [20]	Japan	Cetuximab-Sarotalocan (RM-1929)	Case report	Recurrent oropharyngeal SCC	n = 2	50 J/cm^2^ superficial; 100 J/cm diffuser	Feasibility and safety confirmed; tumor regression in both; manageable local AEs
Kato et al. [21]	Japan	Cetuximab-IR700	Preclinical	EGFR+ mouse models	—	690 nm, 50 J/cm^2^	Tumor shrinkage, dose-dependent response
Inagaki et al. [22]	Japan	Cetuximab-IR700	Preclinical	EGFR+ bone metastasis xenograft (femur)	10 mice (5 treated, 5 control)	50 J/cm^2^; transcutaneous illumination	Selective tumor necrosis in bone; preserved bone structure; proof-of-concept for PIT in osseous lesions
Omura et al. [23]	Japan	Cetuximab-Sarotalocan	Case report	Recurrent nasopharyngeal SCC	n = 1	100 J/cm diffuser (transnasal endoscopic)	Feasible and safe; partial response; no severe AEs
Takao et al. [24]	Japan	Cetuximab-IR700	Preclinical	Hepatocellular Carcinoma	—	690 nm	Tumor growth suppression and selective necrosis demonstrated in HCC models
Shibutani et al. [25]	Japan	Cetuximab-Sarotalocan	Case series	HNSCC	n = 5	690 nm	Post-operative pain management
Nagaya et al. [26]	USA	Cetuximab-IR700	Preclinical	Tripple negative breast cancer xenografts	4–6 mice per group	690 nm	Optimization of conjugate dose and light exposure; selective tumor necrosis demonstrated
Nagaya et al. [27]	USA	Canine anti-EGFR–IR700	Preclinical	Bladder cancer xenograft expressing canine EGFR	~10 mice per group	690 nm	Selective tumor necrosis; proof-of-concept for species-specific EGFR-targeted NIR-PIT
Okamoto et al. [28]	Japan	Cetuximab-Sarotalocan	Case Report	Recurrent HNSCC	n = 2	690 nm	Emergency tracheostomy due to post-PIT airway edema; highlights airway risk and need for careful monitoring
Kushihashi et al. [29]	Japan	Cetuximab-Sarotalocan	Case report	Nasopharyngeal SCC	n = 1	690 nm	Emergency tracheostomy due to post-PIT airway edema; highlights airway risk in nasopharyngeal lesions
Tamagawa et al. [30]	Japan	Cetuximab-Sarotalocan	Case Report	Oropharyngeal SCC	n = 1	690 nm	Salvage PIT feasible after chemoradiotherapy; emphasizes airway monitoring and edema risk
Nishimura et al. [31]	Japan	Cetuximab-Sarotalocan	Case Report	Recurrent Nasopharyngeal SCC	n = 1	690 nm	Lemierre’s syndrome after PIT; highlights rare but serious infectious/vascular complication
Makino et al. [32]	Japan	Cetuximab-Sarotalocan	Case series	Oropharyngeal SCC	n = 6	690 nm	Feasibility and short-term local control in salvage setting; emphasizes airway monitoring
Kado et al. [33]	Japan	Cetuximab-Sarotalocan	Case Report	HNSCC	n = 1	690 nm	Temporary hypocalcemia without hypomagnesemia in patient with hypoparathyroidism; highlights need for electrolyte monitoring
Takuma et al. [34]	Japan	Cetuximab-Sarotalocan	Case Report	Oropharyngeal Cancer	n = 1	690 nm	Severe drug-induced interstitial lung disease after PIT; improved with corticosteroid therapy
Suekata et al. [35]	Japan	Cetuximab-Sarotalocan	Case Report	HNSCC	n = 1	690 nm	Interstitial pneumonia post-PIT; improved with corticosteroid therapy; highlights rare pulmonary toxicity
Ueda et al. [36]	Japan	Cetuximab-Sarotalocan	Prospective Observational	Recurrent / Unresectable HNSCC	n = 7	690 nm; Real-time fluorescence imaging	Real-time fluorescence imaging enabled pharmacokinetic visualization and procedural verification during NIR-PIT
Tanaka et al. [37]	Japan	Cetuximab-IR700 conjugate	Preclinical	Mouse xenograft tumor models	5 mice per group	690 nm	Demonstrated recovery of IR700 fluorescence after PIT; provides mechanistic insight into fluorescence dynamics
Furumoto et al. [38]	Japan	Cetuximab or Panitumumab-IR700	Preclinical	EGFR+ Tumors	5–10 mice per group	690 nm	Tumor selectivity validated
Okamoto et al. [39]	Japan	Cetuximab-Sarotalocan	Retrospective observational study	Recurrent / unresectable HNSCC	n = 40	690 nm	Real-world effectiveness and safety of PIT across multiple Japanese centers
Okada et al. [40]	Japan	Cetuximab-IR700 vs. panitumumab–IR700.	Preclinical	EGFR+ xenograft mouse model	(n = 5–8 mice/group)	50–100 J/cm^2^; single vs. fractionated	Fractionated light improved efficacy; antibody selection influenced outcomes

Among the included works, four were clinical trials, three were observational human studies, eleven were case reports, two were case series, and eight were preclinical experimental studies exploring mechanistic or optimization aspects of NIR-PIT. Most clinical research focused on recurrent or unresectable HNSCC, while several preclinical investigations extended to other EGFR-expressing solid tumors, including bladder, breast cancer and hepatocellular carcinoma using murine xenograft or tumor-bearing animal models.

The photoimmunotherapeutic agent was consistently based on the anti-EGFR monoclonal antibody cetuximab conjugated with the IRDye700DX (IR700) photoabsorber, activated by 690 nm near-infrared light. Illumination parameters commonly ranged between 50–100 J/cm^2^, delivered via superficial or interstitial diffuser systems. Across studies, NIR-PIT was generally reported as technically feasible and well tolerated, with tumor-selective necrosis, manageable local inflammation, and encouraging early tumor control signals. Emerging evidence also highlighted potential workflow barriers, such as light access in anatomically complex regions and coordination between surgical and radiation teams, alongside biological enablers like immunogenic cell death and enhanced tumor perfusion post-irradiation.

## Thematic presentation of barriers and enablers to clinical translation

The 28 included studies collectively represent a translational continuum from mechanistic preclinical investigations to early-phase trials, real-world observational studies, and post-marketing case reports. Preclinical studies focus on optimizing antibody–photoabsorber conjugates, light dosimetry, and mechanistic understanding of treatment effects [22, 24, 26, 27, 37, 38, 40]. Clinical evidence is dominated by HNSCC, spanning early-phase trials [6, 9], combination studies [7], multicentre real-world analyses [39], and multiple case reports and series addressing feasibility and safety in anatomically complex or high-risk settings. Evidence for other solid tumors remains limited, with gastric cancer represented by a single phase Ib combination trial [8] and other tumor types supported largely by preclinical data. Together, these studies identify a set of recurring biological, technical, clinical, and implementation factors that either facilitate or constrain clinical translation (Fig 2).

### Biological and mechanistic determinants

Preclinical studies consistently establish EGFR-targeted NIR-PIT as biologically feasible, with antitumor effects dependent on both adequate target expression and appropriate light exposure. Optimization studies demonstrate that treatment efficacy increases with light dose up to a defined threshold, beyond which additional illumination yields diminishing returns [38, 40]. This non-linear dose–response relationship is a critical translational enabler, as it supports the development of standardized, clinically deliverable light regimens rather than escalation-based strategies. At the same time, reliance on superficial xenograft models introduces a translational barrier, as these systems do not fully recapitulate the optical, vascular, and biological heterogeneity of human tumors.

Mechanistically, NIR-PIT induces rapid and selective tumor cell death following light activation of the antibody-IR700 conjugate, characterized by acute membrane damage and necrotic cell death in EGFR-expressing cells [21, 26]. Preclinical mechanistic studies have further shown that IR700 fluorescence, which decreases immediately after photoactivation, can partially recover over time due to redistribution of intact conjugate rather than treatment failure [37]. The relevance of this fluorescence recovery phenomenon to human tumors remains uncertain, particularly given differences in tissue depth and structure that constrain light delivery in clinical settings [22, 38]. Structural features of tumors, including tissue density and anatomical accessibility, therefore represent important biological barriers to translation. Overall, biological enablers include tumor-selective uptake and reproducible light-dependent cytotoxicity, whereas barriers include potential EGFR heterogeneity, limited validation beyond HNSCC, and uncertain translatability of preclinical mechanistic biomarkers to clinical contexts.

### Technical feasibility, treatment safety, patient selection, and tolerability

Clinical feasibility studies demonstrate that NIR-PIT can be delivered using a range of access routes, including open surgical exposure, endoscopic techniques, and trans nasal illumination. Case reports and series describe successful treatment of recurrent oropharyngeal and nasopharyngeal lesions, including tumors at the base of tongue and lateral pterygoid muscle, which are typically challenging to manage with conventional local therapies [20, 23, 30, 32]. These reports act as translational enablers by illustrating practical solutions to anatomical barriers. However, the same studies underscore persistent technical constraints. Light penetration depth remains a fundamental limitation, restricting applicability to lesions that are superficial or directly accessible. Preclinical work in bone metastasis and hepatocellular carcinoma models confirms that efficacy is contingent on proximity to the illumination source, reinforcing concerns about translation to deep-seated or bulky tumors [22, 24]. These findings collectively position anatomical accessibility as both an enabler and a barrier, depending on tumor location.

Clinical use of NIR-PIT has largely been restricted to recurrent or unresectable EGFR-expressing tumors with lesions that are directly visible or endoscopically accessible, as illustrated in early clinical studies and case series [6, 20]. Quality-of-life analyses have reported improvements in patient-reported outcomes such as pain and swallowing function following treatment, supporting a favorable post-treatment functional profile [18]. Combination therapy with ASP-1929 and pembrolizumab demonstrated a manageable safety profile without unexpected immune-related toxicities, supporting the feasibility of multimodal treatment strategies in advanced disease [7]. Collectively, these findings suggest a favorable therapeutic profile for PIT in selected patients, while highlighting the need for vigilant toxicity monitoring and supportive care protocols.

Despite careful patient selection, treatment-related adverse events remain evident. Early-phase trials and real-world studies report that most adverse events are localized and manageable, safety emerges as a major translational barrier when examined across the full evidence base. Pain, edema, and mucosal inflammation are consistently observed and generally transient, as characterized in focused analyses of post-treatment pain [25] and multicenter real-world cohorts [39]. These effects, while common, are typically controllable and thus function as minor barriers. In contrast, a series of detailed case reports documents rare but severe complications that have significant implications for patient selection and clinical adoption. These include acute airway obstruction requiring emergency tracheostomy following head and neck PIT [28, 29], severe pulmonary toxicities such as interstitial lung disease or pneumonia [34, 35], infectious vascular complications including Lemierre’s syndrome [31], and metabolic disturbances in patients with underlying endocrine disease [33]. Although incidence cannot be inferred from case reports, the recurrence of these events across independent publications suggests that rare but serious toxicities represent a non-trivial barrier to broader translation, particularly outside specialized centers. Importantly, these reports also delineate risk factors and management strategies, thereby functioning as conditional enablers when integrated into refined clinical protocols.

### Clinical workflow and technical adaptability

Across the included studies, clinical workflow integration and technical adaptability emerged as central determinants of successful translation of EGFR-targeted NIR-PIT. Real-world implementation data demonstrate that NIR-PIT can be delivered reproducibly within routine clinical practice when appropriate infrastructure and multidisciplinary coordination are in place. A multicenter retrospective analysis showed consistent safety and effectiveness profiles across institutions, supporting scalability beyond early-phase trial settings and indicating that standardized workflows can be achieved in experienced centers [39]. However, this scalability remains closely linked to the availability of dedicated near-infrared light systems and specialized light delivery devices, which to date have been implemented predominantly within Japanese institutions, reflecting both regulatory and infrastructural constraints.

Several studies highlighted the need for close coordination between surgical, oncology, anesthesiology, and endoscopy teams, particularly in head and neck applications where airway management, patient positioning, and procedural timing are critical. Case series and reports confirmed that NIR-PIT can be successfully integrated into operating rooms and endoscopic suites, but also identified procedural duration, anesthesia requirements, and perioperative airway risk as practical barriers to wider adoption [29]. In anatomically high-risk settings, workflow complexity increased, underscoring the importance of institutional experience and anticipatory planning.

Technical adaptability was demonstrated through the use of navigation systems, endoscopic approaches, and interstitial light delivery to treat lesions in confined or anatomically challenging locations. Navigation-assisted PIT enabled precise light placement in regions with limited access, supporting feasibility in selected cases while also highlighting the need for meticulous mucosal care and post-procedural monitoring [19]. Additional reports described transnasal, endoscopic, and interstitial illumination strategies for nasopharyngeal and base-of-tongue tumors, illustrating how procedural flexibility can partially overcome anatomical constraints [23, 30, 32]. Despite these adaptations, the requirement for direct or interstitial light access remained a persistent barrier, limiting eligibility for patients with deep-seated, diffuse, or poorly accessible disease.

Emerging technological tools further informed workflow optimization. Prospective observational data showed that real-time fluorescence imaging can visualize drug distribution and photobleaching during PIT, providing intra-procedural feedback that may support quality assurance and procedural consistency [36]. When interpreted alongside mechanistic evidence that IR700 fluorescence may recover due to conjugate redistribution rather than treatment failure, these systems offer a framework for cautious interpretation of intra-procedural signals rather than definitive response assessment [37]. While promising, the clinical utility of such imaging for outcome prediction remains unproven.

An additional workflow enabler identified across studies was the repeatability of treatment. Several reports documented the safe administration of repeated PIT sessions at intervals of approximately four to six weeks without evidence of cumulative toxicity, supporting feasibility of retreatment in selected patients [7, 17]. Taken together, these findings indicate that effective clinical workflows for NIR-PIT rely on the integration of specialized equipment, adaptable light delivery strategies, real-time procedural support, and coordinated multidisciplinary care, while ongoing barriers relate to equipment dependency, procedural complexity, and anatomical accessibility.

### Regulatory, reimbursement, and implementation pathways

Regulatory and implementation pathways for EGFR-targeted NIR-PIT are closely linked to its status as a combined drug-device therapy, which necessitates coordinated approval and deployment of both the antibody-photoabsorber conjugate and dedicated light delivery systems. Among the included studies, regulatory maturity is most clearly demonstrated in Japan, where cetuximab sarotalocan sodium (ASP-1929) has progressed from early-phase clinical trials into post-marketing clinical use. This context underlies the predominance of Japanese clinical evidence, including multicenter retrospective analyses and post-marketing safety reports [6, 39].

Implementation data from real-world Japanese cohorts show that, within an established regulatory framework, NIR-PIT can be delivered reproducibly across institutions using standardized protocols and dedicated equipment [39]. Multiple case reports and series further illustrate that PIT has been integrated into routine practice in operating rooms and endoscopic suites, supporting feasibility beyond controlled trial environments [20, 32]. However, the reliance on proprietary near-infrared light systems and specialized light guides is implicit across all clinical studies, representing a structural requirement for treatment delivery rather than an optional component [6, 39].

Outside Japan, regulatory progress remains limited to investigational use. Early-phase trials conducted in the United States evaluated ASP-1929 under strictly protocolized conditions, with narrowly defined eligibility criteria and intensive safety monitoring, reflecting the regulatory caution associated with a novel drug-device modality [7, 9]. The absence of large confirmatory trials or real-world implementation studies in these settings indicates that broader regulatory approval and reimbursement pathways have not yet been established.

Reimbursement considerations are not explicitly analyzed in the included studies. While not directly examined, these structural requirements have implications for reimbursement and health-system integration. Nonetheless, implementation-level evidence suggests that treatment delivery requires substantial institutional resources, including specialized equipment, trained multidisciplinary teams, and procedural infrastructure. The concentration of real-world experience within high-volume centers and the lack of data from community or resource-limited settings imply that reimbursement and health-system integration remain unresolved barriers to wider dissemination [39].

### Evidence gaps and future priorities

Despite growing clinical experience, the included studies reveal several important evidence gaps that limit further translation of EGFR-targeted NIR-PIT. Most notably, clinical evidence remains heavily concentrated in head and neck squamous cell carcinoma. While early-phase trials and real-world studies demonstrate feasibility and safety in this setting [6, 39], translation to other solid tumors is limited. Gastric cancer is represented by a single phase Ib combination study [8], while other tumor types, including breast cancer, bladder cancer, hepatocellular carcinoma, and bone metastases, are supported primarily by preclinical investigations [22, 24, 26, 27]. This imbalance highlights the need for carefully designed clinical studies to validate preclinical feasibility in non-HNSCC populations.

Another key gap concerns patient selection and predictive biomarkers. Although EGFR expression is a prerequisite for treatment, few clinical studies systematically assess expression heterogeneity or correlate molecular features with treatment response. Preclinical and observational studies exploring fluorescence dynamics and real-time imaging provide mechanistic insight into drug distribution and photobleaching [36, 37], but none establish these parameters as predictive biomarkers of clinical outcome. As such, the clinical relevance of mechanistic imaging findings remains uncertain.

Safety characterization represents an additional priority. While early-phase trials and real-world studies report predominantly localized and manageable adverse events [6, 39], multiple case reports document rare but serious complications, including airway obstruction requiring emergency tracheostomy, pulmonary toxicity, infectious vascular events, and metabolic disturbances in susceptible patients [28, 29, 31, 33–35]. These reports underscore the need for improved risk stratification, refined eligibility criteria, and standardized toxicity monitoring protocols in future studies.

Methodologically, the current evidence base is dominated by early-phase trials, retrospective analyses, and case reports. Comparative studies evaluating NIR-PIT against existing local or systemic therapies are lacking, limiting conclusions regarding relative benefit. Long-term outcomes, including durability of local control, functional preservation, and survival, are inconsistently reported across studies and remain insufficiently characterized in real-world populations [39].

Finally, implementation and health-system factors remain underexplored. None of the included studies formally assess cost-effectiveness, learning curves, or scalability across diverse healthcare settings. Given the equipment-dependent nature of NIR-PIT and its reliance on specialized expertise, future research should incorporate implementation-focused and health-economic analyses to support broader clinical adoption.

## Discussion

To our knowledge, this is the first review to systematically synthesize barriers and enablers to the clinical translation of EGFR-targeted NIR-PIT by explicitly identifying biological, technical, clinical, and system-level barriers and enablers shaping its clinical adoption. Although the biological principles and early clinical applications of NIR-PIT have been well described, existing review literature has largely focused on mechanistic rationale, proof-of-concept efficacy, or disease-specific clinical outcomes rather than on translational determinants. As a result, the present work addresses an important gap by reframing the emerging evidence base through an implementation-oriented lens.

From a biological perspective, the included preclinical studies consistently demonstrate that EGFR-targeted NIR-PIT induces rapid, selective tumor cell death following light activation of the antibody-IR700 conjugate. This process, characterized by acute membrane disruption and necrotic cell death, represents a key enabler by distinguishing NIR-PIT from conventional photodynamic therapy and systemic EGFR inhibition. Other studies have identified this mechanistic specificity, together with the potential for immunogenic cell death, as a defining feature of the platform [41, 42]. Importantly, NIR-PIT using antibody-IR700 conjugates falls within the broader conceptual framework of “AntibodyPlus” therapeutics, a growing category of hybrid modalities that combine an antibody component with an effector module to enhance therapeutic activity. Zhu and colleagues formally defined and systematized this framework [43], situating photoimmunotherapy alongside other conjugate and combination strategies such as antibody-drug conjugates, bispecific antibodies, and antibody-radioisotope conjugates. Recognizing NIR-PIT within the AntibodyPlus paradigm provides a useful conceptual lens: the antibody component (e.g. cetuximab) confers target specificity and favourable pharmacokinetics, while the IR700 photoabsorber module enables spatially controllable cell killing. This framing also invites comparisons with related modalities and highlights the shared challenges of conjugate stability, payload delivery, and manufacturing scalability that are common across the AntibodyPlus class. However, the present synthesis demonstrates that while immunologically relevant effects are well described in preclinical and conceptual literature, direct clinical evidence of systemic immune activation remains limited within the included human studies, constraining claims regarding immune-mediated benefit.

Optimization studies within the synthesis further identify a non-linear relationship between light dose and therapeutic efficacy, with diminishing returns beyond defined exposure thresholds. This finding functions as a translational enabler by supporting standardized, clinically deliverable light regimens rather than escalation-based strategies. Similar conclusions have been advanced in a review, which caution against indiscriminate dose escalation in photoimmunotherapy due to photophysical and biological constraints [41]. At the same time, reliance on superficial xenografts and simplified tumor geometry may overestimate uniform light exposure and underestimate the impact of human tumor heterogeneity (optical properties, vascularity, stromal density). Clinically, this matters because adequate target expression does not guarantee adequate light exposure, which is a recurring theme in translational discussions of PIT and related optical therapies [44]. In addition, both the included studies and prior reviews emphasize that tumor depth, stromal density, and vascular heterogeneity in human cancers substantially alter light propagation and antibody distribution, limiting direct extrapolation of preclinical efficacy to clinical settings [41].

A critical and mechanistically distinctive feature of NIR-PIT, one that distinguishes it from conventional photodynamic therapy and merits elaboration beyond its role as a local cytotoxic modality, is its capacity to induce immunogenic cell death (ICD) and, potentially, systemic immune activation including abscopal effects. In NIR-PIT, rapid and selective disruption of tumor cell membranes by photoactivated IR700 releases damage-associated molecular patterns, tumor-associated antigens, and pro-inflammatory mediators, collectively creating a pro-immunogenic microenvironment. Preclinical evidence has consistently demonstrated that NIR-PIT-induced ICD can prime dendritic cells, augment CD8+ cytotoxic T lymphocyte activity, and generate systemic anti-tumor immune responses that extend beyond the illuminated lesion [5, 41, 42]. The abscopal effect, regression of non-irradiated, distant tumors following local treatment, has been documented in murine models of NIR-PIT and is attributed to this systemic immune priming. In the clinical context, the potential for abscopal responses is a key rationale for combining NIR-PIT with systemic immunotherapy, particularly immune checkpoint inhibitors. The combination of ASP-1929 with pembrolizumab demonstrated manageable safety in an early-phase trial [7], reflecting the hypothesis that PIT-induced immune activation could be amplified and sustained by concurrent checkpoint blockade. Similarly, combination with nivolumab was evaluated in gastric cancer [8], further extending this translational rationale beyond HNSCC. Despite this promising biological framework, direct clinical evidence of systemic immune activation or abscopal responses in human PIT studies remains limited. Most included clinical studies were not designed to detect distant responses or systematically assess immunological endpoints, leaving the magnitude and durability of immune-mediated effects uncertain in the human setting. Future trials should incorporate immunological correlates (e.g. circulating T cell phenotyping, cytokine profiling, tumor-infiltrating lymphocyte assessment in untreated lesions) as exploratory endpoints to substantiate and quantify the abscopal and systemic immune effect.

Clinically, translation has been most advanced in HNSCC, where tumor accessibility and established multidisciplinary workflows act as major enablers. This pattern aligns with systematic review evidence identifying HNSCC as the most suitable clinical entry point for NIR-PIT due to anatomical exposure and unmet needs in recurrent disease [39]. Case reports and series within the synthesis further demonstrate technical adaptability through endoscopic, transnasal, navigation-assisted, and interstitial illumination strategies, illustrating how procedural innovation can mitigate anatomical barriers. Our study clearly demonstrates that light penetration depth is a hard gatekeeper, and preclinical work in deeper or less accessible disease (e.g. liver, bone models) reinforces that efficacy depends strongly on proximity to the light source. This is widely recognized in broader PIT literature as one of the fundamental determinants of which indications are naturally compatible with the technology versus those that require major device innovation [45]. Nevertheless, both the included studies and broader review literature consistently identify the requirement for direct or interstitial light access as a fundamental limitation, restricting applicability in deep-seated, bulky, or diffusely infiltrative tumors [39].

Safety considerations emerge as a critical and nuanced determinant of translation. Across early-phase trials and real-world cohorts, most adverse events are localized and manageable, supporting a favourable tolerability profile relative to many cytotoxic or ablative therapies. Reviews of NIR-PIT similarly emphasize its tumor-selective toxicity as a key advantage [4]. However, the synthesis of case-based evidence reveals a recurring pattern of rare but severe complications, including airway compromise, pulmonary toxicity, infectious vascular events, and metabolic disturbances. Importantly, systematic and narrative reviews caution that such events are likely under-recognized during early adoption phases and underscore the need for refined patient selection and structured post-marketing surveillance [39]. These findings position safety not as a binary enabler or barrier, but as a conditional factor dependent on institutional expertise and patient risk stratification. Edema represents one of the most clinically significant and recurrent adverse events associated with ASP-1929 PIT, and its mechanistic basis has recently begun to be elucidated. Preclinical data presented by Rakuten Medical at the American Association for Cancer Research (AACR) Annual Meeting 2026 demonstrated that photoimmunotherapy induces the upregulation of cyclooxygenase-2 (COX-2) expression followed by prostaglandin E₂ (PGE₂) secretion, which drives vascular permeabilization and edema formation. Prophylactic administration of both selective COX-2 inhibitors (robenacoxib, meloxicam) and pan-COX inhibitors (flurbiprofen) significantly reduced PIT-induced edema, by approximately 30–50%, in syngeneic mouse tumor models without compromising anti-tumor efficacy [46].

Workflow integration and technical adaptability further shape translational readiness. Multicenter real-world experience demonstrates that NIR-PIT can be incorporated into routine clinical practice within specialized centers equipped with dedicated light systems and coordinated multidisciplinary teams. Real-time fluorescence imaging and navigation systems are increasingly explored as procedural adjuncts to support light delivery and intra-procedural assessment. However, these technologies currently function as supportive tools rather than validated predictors of treatment response. While real-time fluorescence monitoring may support procedural consistency, mechanistic findings caution against over-interpreting fluorescence recovery as a proxy for failure, thereby reinforcing the idea that imaging currently fits best as intra-procedural support/quality assurance rather than as a definitive predictive biomarker [47].

Finally, regulatory and health-system factors represent overarching barriers to broader dissemination. The geographic concentration of post-marketing and real-world experience in Japan is therefore interpretable as regulatory maturity plus infrastructure availability, rather than mere publication clustering. Secondary clinical summaries explicitly note Japan’s approval context for cetuximab sarotalocan-based PIT, the dedicated 690-nm laser system, and the national health insurance coverage for the treatment of patients with EGFR-expressed HNSCC [48, 49]. Outside Japan, the evidence remains largely investigational and protocolized, which is consistent with earlier-stage reimbursement and service-line integration. IR700 access is a practical translational constraint for PIT development. Historically, IR700 NHS ester was commercially available from LI-COR Biosciences for research and conjugation purposes. However, in December 2020, Rakuten Medical, Inc. acquired the intellectual property, manufacturing technology, marketing rights, and all associated data for IR700 from LI-COR, establishing an exclusive position as its sole owner, manufacturer, and global supplier [53, 54]. This consolidation of the IR700 supply chain has significant implications: academic institutions, independent research groups, and biopharmaceutical companies seeking to develop novel IR700-based conjugates, including those for EGFR-targeted PIT, must now negotiate access with Rakuten Medical rather than procuring from an open commercial market. In January 2026, Rakuten Medical announced expanded academic access to IR700 at an administrative fee-only acquisition cost with increased flexibility for publication, intellectual property ownership, and commercialisation, thereby aiming to accelerate open innovation [50, 51]. Nonetheless, for commercial entities and translational research programmes, supply security, royalty considerations, and IP landscape remain non-trivial structural constraints. These supply-chain dynamics interact directly with the translational barriers discussed in this review and should be recognized as a distinct, technology-level consideration when evaluating the feasibility of scaling IR700-based PIT, whether for ASP-1929 or novel constructs being developed by other organisations.

### Emerging EGFR-targeted photoimmunotherapy programs: NIRa biosciences and Daiichi Sankyo

Although cetuximab sarotalocan (ASP-1929) remains the only photoimmunotherapy construct to have entered clinical practice, several emerging programmes are advancing novel antibody-photosensitizer conjugates that target EGFR and are therefore directly relevant to the translational landscape described in this review. Two leading programmes warrant specific discussion.

NIRa Biosciences is a biotechnology company developing next-generation photosensitizer conjugates for photoimmunotherapy, with a platform based on benzoporphyrin analogues, a class of photosensitizers distinct from IR700. Patent WO2024216150A1 (NIRa Biosciences Inc., inventors Sadiki, Zhou, and Liu) discloses benzoporphyrin analogue compounds and their conjugates for use in photodynamic and photoimmunotherapy applications, including EGFR-targeted constructs [52]. The benzoporphyrin scaffold offers potentially distinct photophysical properties compared to IR700, including absorption profiles that may permit different tissue penetration or activation characteristics. The significance of this programme for the present review is twofold: first, it demonstrates that the antibody-photosensitizer conjugate space is expanding beyond IR700, with multiple chemical scaffolds under investigation. Second, it raises questions about comparative safety and efficacy profiles relative to the IR700-based platform that has been the exclusive focus of clinical experience to date. As this programme is at early preclinical/patent stage, its clinical translatability and side-effect profile, including whether alternative photosensitizers would confer different or reduced edema risk, remain to be established.

Daiichi Sankyo, a major Japanese pharmaceutical company with an established expertise in antibody-drug conjugates (ADCs), has filed patent WO2025105486A1 (Daiichi Sankyo Co. Ltd) disclosing novel antibody-photosensitive substance conjugates [53]. Daiichi Sankyo’s entry into the antibody-photosensitizer conjugate space is notable given the company’s extensive ADC manufacturing capabilities and commercial experience. The convergence of ADC and photoimmunotherapy platforms by a company of this scale suggests growing industry recognition of photoimmunotherapy as a viable drug-conjugate modality. It also indicates that the technical and manufacturing challenges of antibody-photosensitizer conjugation, which share many parallels with ADC production, are amenable to established industrial processes. Both programmes reinforce the broader message of this review that the photoimmunotherapy landscape is actively evolving, and that cetuximab sarotalocan, whilst clinically pioneering, represents the first rather than the last antibody-photosensitizer conjugate to reach clinical development.

### IR700-based photoimmunotherapy beyond EGFR: aura biosciences and virus-like drug conjugates

A further IR700-based photoimmunotherapy programme of direct relevance to this review is being developed by Aura Biosciences, a clinical-stage oncology company that has applied the IR700 dye to a proprietary Virus-Like Drug Conjugate (VDC) platform. Rather than conjugating IR700 to a monoclonal antibody, Aura Biosciences uses virus-like particles, non-replicating, self-assembling protein shells derived from human papillomavirus capsid proteins, as a targeting vehicle to which IR700 is conjugated. These VDCs are designed to selectively bind to heparan sulphate proteoglycans (HSPGs) that are overexpressed on the surface of certain tumour types, enabling targeted near-infrared photoimmunotherapy via a mechanism analogous to, but distinct from, antibody-based PIT [54].

Aura Biosciences’ lead candidate, belzupacap sarotalocan (bel-sar, formerly AU-011), is the most advanced IR700-based photoimmunotherapy programme outside the EGFR-cetuximab sarotalocan platform, with bel-sar now in the global Phase 3 CoMpass trial for first-line treatment of adults with primary indeterminate lesions or small choroidal melanoma. Aura describes bel-sar as its lead VDC candidate and reports that the CoMpass trial is randomized, masked, sham-controlled and intended to support treatment of early-stage choroidal melanoma, a rare ocular cancer with no approved targeted or vision-preserving drug therapy in this setting [55]. Phase 2 data reported 80% tumour control and 90% visual acuity preservation among Phase 3-eligible patients, while the platform has also expanded into non–muscle-invasive bladder cancer using cystoscopic focal administration with laser activation [56].

The Aura Biosciences programme is relevant to this review in two respects. First, it provides an important proof-of-concept that IR700 can serve as a therapeutic photoabsorber in conjugate systems beyond antibodies, with demonstrable clinical activity in diseases beyond HNSCC. Second, it raises a key translational question that remains unanswered: whether IR700 causes similar side effects, particularly the oedema, inflammation, and rare severe complications documented in the ASP-1929 literature, in other anatomical and disease contexts. The intravitreal and intravesical routes of bel-sar administration differ substantially from the locoregional or endoscopic illumination routes used in head and neck PIT, and the adverse event profiles may therefore differ. Comparative analysis of IR700-related toxicities across different conjugate-target-tissue combinations will be an important area for future research. Overall, the existence of a Phase 3 IR700-based photoimmunotherapy programme strongly validates the clinical potential of the IR700 photoabsorber platform and broadens the implications of this review beyond EGFR-targeted applications.

## Recommendations for clinical practice

- Clinical use of EGFR-targeted NIR-PIT should remain confined to specialized centers with appropriate regulatory approval, dedicated near-infrared systems, and multidisciplinary expertise, particularly for head and neck malignancies.
- Careful patient selection and proactive toxicity monitoring are essential, especially in anatomically high-risk settings or in patients with significant comorbidities.
- Real-time imaging and navigation tools should be used as procedural aids, not as surrogate efficacy markers, until validated in prospective clinical studies.

## Recommendation for future research

- Prospective comparative trials are needed to define the relative benefit of NIR-PIT compared with established local and systemic therapies across patient-centered outcomes.
- Expand mechanistic research into immune-competent and heterogeneous tumor models, addressing limitations identified in primary studies.
- Incorporate immunological correlates (e.g. circulating T cell phenotyping, cytokine profiling, tumor-infiltrating lymphocyte assessment in non-treated lesions) as exploratory endpoints to substantiate and quantify the abscopal and systemic immune effects that are well-characterized preclinically.
- Explore additional antigenic targets and combination strategies, particularly immune-modulatory targets, to broaden the applicability of NIR-PIT beyond EGFR.
- Implementation science and health-economic analyses should be integrated into future clinical programs to support reimbursement and policy decisions.

## Strengths and limitations

Inclusion of diverse study designs enabled capture of mechanistic insights, implementation challenges, and rare but clinically significant adverse events that are often not represented in controlled trials. Methodological rigor was supported through the use of appropriate quality appraisal tools to appraise the quality of included studies according to design, informing interpretation while maintaining the inclusive nature of a scoping review. However, the evidence base remains heavily weighted toward head and neck squamous cell carcinoma and toward early-phase studies, retrospective analyses, and case reports, limiting the generalisability and precluding quantitative synthesis. Heterogeneity in study design, intervention protocols, outcome reporting, and follow-up further constrained comparative interpretation. In addition, regulatory, reimbursement, and health-economic considerations were not directly evaluated in the included studies, restricting conclusions regarding scalability and system-level adoption. These limitations reflect gaps in the underlying literature rather than deficiencies in the review process and highlight priorities for future research.

## Conclusion

This scoping review synthesizes current evidence on the barriers and enablers to clinical translation of EGFR-targeted NIR-PIT across advanced solid tumors. The findings demonstrate that NIR-PIT is supported by a strong biological rationale and has achieved meaningful clinical translation in selected settings, particularly recurrent or unresectable HNSCC, where anatomical accessibility, established workflows, and regulatory approval have facilitated adoption. However, translation beyond this niche remains constrained by fundamental limitations related to light delivery, anatomical accessibility, rare but serious toxicities, equipment dependency, and an uneven regulatory and reimbursement landscape. While emerging technical adaptations, real-time imaging tools, and combination strategies represent important enablers, the current evidence base is dominated by early-phase studies, case reports, and retrospective analyses, with limited comparative or long-term outcome data. Addressing these gaps through carefully designed clinical trials, improved patient selection strategies, and implementation-focused research will be essential to advance NIR-PIT from a specialized intervention toward broader, evidence-based clinical integration.

## Animal research statement

Not applicable.

## Supplementary Material

Supplementary_materials_tbag026

## Data Availability

The original contributions presented in the study are included in the article/supplementary material, further enquiries can be directed to the corresponding author.

## References

[1] Fu Z, Li S, Han S. et al. Antibody drug conjugate: the “biological missile” for targeted cancer therapy. *Signal Transduct Target Ther* 2022;7:93. 10.1038/s41392-022-00947-7. 35318309 PMC8941077

[2] Kalyankrishna S, Grandis JR. Epidermal growth factor receptor biology in head and neck cancer. *J Clin Oncol* 2006;24:2666–72. 10.1200/jco.2005.04.8306. 16763281

[3] Mitsunaga M, Ogawa M, Kosaka N. et al. Cancer cell–selective in vivo near infrared photoimmunotherapy targeting specific membrane molecules. *Nat Med* 2011;17:1685–91. 10.1038/nm.2554. 22057348 PMC3233641

[4] Ailioaie LM, Ailioaie C, Litscher G. Next-generation cancer treatment: Photoimmunotherapy’s promise for Unresectable head and neck cancers. *Pharmaceutics* 2025;17:716. 10.3390/pharmaceutics17060716. 40574027 PMC12196017

[5] Ogawa M, Tomita Y, Nakamura Y. et al. Immunogenic cancer cell death selectively induced by near infrared photoimmunotherapy initiates host tumor immunity. *Oncotarget* 2017;8:10425–36. 10.18632/oncotarget.14425. 28060726 PMC5354669

[6] Tahara M, Okano S, Enokida T. et al. A phase I, single-center, open-label study of RM-1929 photoimmunotherapy in Japanese patients with recurrent head and neck squamous cell carcinoma. *Int J Clin Oncol* 2021;26:1812–21. 10.1007/s10147-021-01960-6. 34165660 PMC8449763

[7] Cognetti DM, Curry JM, Johnson J. et al. Safety and efficacy findings from a phase Ib II study of ASP-1929 Photoimmunotherapy with Pembrolizumab in recurrent and/or metastatic head and neck squamous cell carcinoma. *Head Neck* 2025;48:160–74. 10.1002/hed.70014. 40852760 PMC12703558

[8] Kadota T, Fujii R, Koyama S. et al. A phase Ib study of photoimmunotherapy with ASP-1929 in combination with nivolumab for advanced gastric cancer (GE-PIT, EPOC1901). *Gastric Cancer* 2025;28:718–24. 10.1007/s10120-025-01623-9. 40372585

[9] Cognetti DM, Johnson JM, Curry J. et al. Phase 1/2a, open-label, multicenter study of RM-1929 photoimmunotherapy in patients with locoregional, recurrent head and neck squamous cell carcinoma. *Head Neck* 2021;43:3875–87. 10.1002/hed.26885. 34626024 PMC9293150

[10] National Cancer Institute (NCI) . *Japan Approves Photoimmunotherapy for Head and Neck Cancer | Center for Cancer Research*. Cancer.gov, https://ccr.cancer.gov/news/article/japan-approves-photoimmunotherapy-for-head-and-neck-cancer?utm_source Accessed 26.01.04.

[11] Tricco AC, Lillie E, Zarin W. et al. PRISMA extension for scoping reviews (PRISMA-ScR): checklist and explanation. *Ann Intern Med* 2018;169:467–73. 10.7326/M18-0850. 30178033

[12] Pollock D, Peters MDJ, Khalil H. et al. Recommendations for the extraction, analysis, and presentation of results in scoping reviews. *JBI Evid Synth* 2023;21:520–32. 10.11124/jbies-22-00123. 36081365

[13] Cochrane Bias Method Group . *Risk of Bias Tools*—*Original (2016) Version of ROBINS-I*. Google.com, https://sites.google.com/site/riskofbiastool/welcome/home/original-2016-version-of-robins-i Accessed 26.02.13.

[14] CASP . *Critical Appraisal Checklists*. Critical Appraisal Skills Programme, https://casp-uk.net/casp-tools-checklists/ (26 February 2009, date last accessed).

[15] Joanna Briggs Institute (JBI) . *Critical Appraisal Tools*. JBI, https://jbi.global/critical-appraisal-tools (26 February 2009, date last accessed).

[16] Hooijmans CR, Rovers MM, de Vries RB. et al. SYRCLE’s risk of bias tool for animal studies. *BMC Med Res Methodol* 2014;14:43. 10.1186/1471-2288-14-43. 24667063 PMC4230647

[17] Okamoto I, Okada T, Tokashiki K. et al. Photoimmunotherapy for managing recurrent laryngeal cancer cervical lesions: a case report. *Case Rep Oncol* 2022;15:34–9. 10.1159/000521435. 35221967 PMC8832182

[18] Okamoto I, Okada T, Tokashiki K. et al. Quality-of-life evaluation of patients with Unresectable locally advanced or locally recurrent head and neck carcinoma treated with head and neck Photoimmunotherapy. *Cancers* 2022;14:4413. 10.3390/cancers14184413. 36139573 PMC9496661

[19] Okamoto I, Okada T, Tokashiki K. et al. Case treated with Photoimmunotherapy under a navigation system for recurrent lesions of the lateral pterygoid muscle. *In Vivo* 2022;36:1035–40. 10.21873/invivo.12799. 35241568 PMC8931901

[20] Nishikawa D, Suzuki H, Beppu S. et al. Near-infrared Photoimmunotherapy for oropharyngeal cancer. *Cancers* 2022;14:5662–2. 10.3390/cancers14225662. 36428754 PMC9688155

[21] Kato T, Furusawa A, Okada R. et al. Near-infrared Photoimmunotherapy targeting Podoplanin-expressing cancer cells and cancer-associated fibroblasts. *Mol Cancer Ther* 2022;22:75–88. 10.1158/1535-7163.mct-22-0313. PMC9812859 36223542

[22] Inagaki FF, Wakiyama H, Aki Furusawa A. et al. Near-infrared photoimmunotherapy (NIR-PIT) of bone metastases. *Biomed Pharmacother* 2023;160:114390–0. 10.1016/j.biopha.2023.114390. 36791566 PMC10024949

[23] Omura G, Honma Y, Matsumoto Y. et al. Transnasal photoimmunotherapy with cetuximab sarotalocan sodium: outcomes on the local recurrence of nasopharyngeal squamous cell carcinoma. *Auris Nasus Larynx* 2023;50:641–5. 10.1016/j.anl.2022.06.004. 35779979

[24] Takao S, Fukushima H, King AP. et al. Near-infrared photoimmunotherapy in the models of hepatocellular carcinomas using cetuximab-IR700. *Cancer Sci* 2023;114:4654–63. 10.1111/cas.15965. 37817415 PMC10727998

[25] Shibutani Y, Sato H, Suzuki S. et al. A case series on pain accompanying Photoimmunotherapy for head and neck cancer. *Healthcare* 2023;11:924–4. 10.3390/healthcare11060924. 36981581 PMC10048590

[26] Nagaya T, Sato K, Harada T. et al. Near infrared Photoimmunotherapy targeting EGFR positive triple negative breast cancer: optimizing the conjugate-light regimen. *PloS One* 2015;10: e0136829. 10.1371/journal.pone.0136829. 26313651 PMC4552472

[27] Nagaya T, Okuyama S, Ogata F. et al. Near infrared photoimmunotherapy targeting bladder cancer with a canine anti-epidermal growth factor receptor (EGFR) antibody. *Oncotarget* 2018;9:19026–38. 10.18632/oncotarget.24876. 29721181 PMC5922375

[28] Okamoto I, Okada T, Tokashiki K. et al. Two cases of emergency tracheostomy after head and neck Photoimmunotherapy. *Cancer Diagn Progn* 2024;4:85–90. 10.21873/cdp.10291. 38173663 PMC10758850

[29] Kushihashi Y, Masubuchi T, Okamoto I. et al. A case of Photoimmunotherapy for nasopharyngeal carcinoma requiring emergency tracheostomy. *Case Rep Oncol* 2024;17:471–6. 10.1159/000537898. 38500712 PMC10948172

[30] Tamagawa S, Okuda K, Nishikawa D. et al. Near infrared Photoimmunotherapy for Locoregionally recurrent oropharyngeal squamous cell carcinoma at Tongue Base after Chemoradiotherapy: a case report and literature review. *Cureus* 2024;16:e74742. 10.7759/cureus.74742. 39735016 PMC11682689

[31] Nishimura M, Okamoto I, Ito T. et al. Lemierre’s syndrome after head and neck Photoimmunotherapy for local recurrence of nasopharyngeal carcinoma. *Case Rep Oncol* 2024;17:180–5. 10.1159/000535597. 38304554 PMC10834035

[32] Makino T, Nishikori A, Sato Y. et al. Near-infrared photoimmunotherapy for recurrent cancer at the base of the tongue. *Photodiagnosis Photodyn Ther* 2025;54:104719–9. 10.1016/j.pdpdt.2025.104719. 40659300

[33] Kado M, Saito Y, Sakamoto T. et al. Temporary hypocalcemia induced by cetuximab sarotalocan without hypomagnesemia in a patient with hypoparathyroidism: a novel case report. *J Pharm Health Care Sci* 2025;11:58. 10.1186/s40780-025-00465-y. 40615905 PMC12228235

[34] Takuma S, Karayama M, Suzuki R. et al. Severe drug-induced interstitial lung disease after photoimmunotherapy with cetuximab–sarotalocan sodium in a patient with oropharyngeal cancer: a case report. *Respir Med Case Rep* 2025;57:102260–0. 10.1016/j.rmcr.2025.102260. 40688713 PMC12275018

[35] Suekata Y, Otsuka T, Asai T. et al. Interstitial pneumonia caused by Photoimmunotherapy with cetuximab-IR700 conjugate. *Auris Nasus Larynx* 2025;52:394–7. 10.1016/j.anl.2025.06.008. 40889202

[36] Ueda T, Taruya T, Chikuie N. et al. Evaluation of real-time pharmacokinetics using fluorescence imaging system in near-infrared photoimmunotherapy for head and neck cancer. *Auris Nasus Larynx* 2025;52:346–53. 10.1016/j.anl.2025.05.001. 40424829

[37] Tanaka H, Okuyama S, Shirota K. et al. Recovery of IR700 fluorescence after near-infrared Photoimmunotherapy: discovery and mechanistic insights. *Cancers* 2026;18:162. 10.3390/cancers18010162. 41514670 PMC12784890

[38] Furumoto H, Okada R, Kato T. et al. Optimal light dose for hEGFR-targeted near-infrared Photoimmunotherapy. *Cancers* 2022;14:4042. 10.3390/cancers14164042. 36011036 PMC9406827

[39] Okamoto I . Photoimmunotherapy for head and neck cancer: a systematic review. *Auris Nasus Larynx* 2025;52:186–94. 10.1016/j.anl.2025.01.005. 39951877

[40] Okada R, Kato T, Furusawa A. et al. Selection of antibody and light exposure regimens alters therapeutic effects of EGFR-targeted near-infrared photoimmunotherapy. *Cancer Immunol Immunother* 2022;71:1877–87. 10.1007/s00262-021-03124-x. 35013765 PMC9271517

[41] Kobayashi H, Choyke PL. Near-infrared Photoimmunotherapy of cancer. *Acc Chem Res* 2019;52:2332–9. 10.1021/acs.accounts.9b00273. 31335117 PMC6704485

[42] Kobayashi H, Furusawa A, Rosenberg A. et al. Near-infrared photoimmunotherapy of cancer: a new approach that kills cancer cells and enhances anti-cancer host immunity. *Int Immunol* 2020;33:7–15. 10.1093/intimm/dxaa037. PMC7771006 32496557

[43] Zhu Y, Wang SS, Zhou ZS. et al. The emergence of AntibodyPlus: the future trend of antibody-based therapeutics. *Antib Ther* 2022;5:280–7. 10.1093/abt/tbac024. 36299417 PMC9590318

[44] Paraboschi I, Turnock S, Kramer-Marek G. et al. Near-InfraRed PhotoImmunoTherapy (NIR-PIT) for the local control of solid cancers: challenges and potentials for human applications. *Crit Rev Oncol Hematol* 2021;161:103325–5. 10.1016/j.critrevonc.2021.103325. 33836238 PMC8177002

[45] Maruoka Y, Wakiyama H, Choyke PL. et al. Near infrared photoimmunotherapy for cancers: a translational perspective. *EBioMedicine* 2021;70:103501. 10.1016/j.ebiom.2021.103501. 34332294 PMC8340111

[46] Amantea CM, Lozano A, Takahashi R. et al. COX-2/PGE2-driven, photoimmunotherapy-induced edema is reduced by prophylactic NSAIDs in mouse tumor models. *Cancer Res* 86:4297. 10.1158/1538-7445.AM2026-4297.

[47] Monaco H, Yokomizo S, Choi HS. et al. Quickly evolving near-infrared photoimmunotherapy provides multifaceted approach to modern cancer treatment. *VIEW* 2021;3:20200110. 10.1002/viw.20200110. 37448778 PMC10343942

[48] Allen D, Szoo MJ, van Bergen TD. et al. Near-infrared Photoimmunotherapy: mechanisms, applications, and future perspectives in cancer research. *Antib Ther* 2025;8:68–85. 10.1093/abt/tbaf001. 39958565 PMC11826922

[49] Okada R, Asakage T. Near-infrared photoimmunotherapy: basics and clinical application. *Jpn J Clin Oncol* 2025;55:843–51. 10.1093/jjco/hyaf069. 40319478

[50] Rakuten Medical, Inc . Rakuten medical completes technology transfer of IR700 manufacturing and establishes exclusive position in its supply. Press release. 4 February 2025. Available at: https://rakuten-med.com/us/news/press-releases/2025/02/04/7883/

[51] Rakuten Medical, Inc . Rakuten medical expands academic access to IR700 dye through fee-only provision, greater publication freedom and expanded IP & commercialisation opportunities. Press release. January 20, 2026. Available at: https://www.prnewswire.com/news-releases/rakuten-medical-expands-academic-access-to-ir700-dye-302665130.html

[52] Sadiki A, Zhou ZS, Liu S. *Benzoporphyrin Analogs, Conjugates, and Methods of Use Thereof*. International Patent WO2024216150A1. NIRa Biosciences Inc. Filed April 2024, 2024. Available at:, https://patents.google.com/patent/WO2024216150A1/.

[53] Iwashiro T, Meguro M, Ota I. et al. *Antibody-Photosensitive Substance Conjugate*. International Patent WO2025105486A1. Daiichi Sankyo Co. Ltd. Filed November 2024, 2025. Available at:, https://patents.google.com/patent/WO2025105486A1/en.

[54] Kines RC, Varsavsky I, Choudhary S. et al. An infrared dye-conjugated virus-like particle for the treatment of primary uveal melanoma. *Mol Cancer Ther* 2018;17:565–74. 10.1158/1535-7163.MCT-17-0953. 29242243 PMC8063570

[55] Aura Biosciences, Inc . CoMpass phase 3 trial of belzupacap sarotalocan (bel-Sar) for small choroidal melanoma. Company pipeline Available at: https://www.aurabiosciences.com (April 2026, date last accessed).

[56] Aura Biosciences, Inc . *Aura Biosciences Reports Positive Phase 2 End of Study Results Evaluating Bel-Sar as a First-Line Treatment for Early-Stage Choroidal Melanoma*—*Aura Biosciences*. Aura Biosciences, https://ir.aurabiosciences.com/news-releases/news-release-details/aura-biosciences-reports-positive-phase-2-end-study-results (26 April 2027, date last accessed).

