# Supplementary material for: Epidermal growth factor receptor–targeted near-infrared photoimmunotherapy for epidermal growth factor receptor–expressing advanced solid tumors: a scoping review of barriers and enablers to clinical translation
Source: Antib Ther. 2026 Jun 5;9(3):407–19. doi: 10.1093/abt/tbag026 (PMC13463628; doi:10.1093/abt/tbag026)
Supplement: Supplementary file 1 [file supplementary_materials_tbag026.doc]

**Appendix 1.**

PubMed

| Concepts | Search string | Results (2015 – 2026) |
| --- | --- | --- |
| #1  Tumour | (“Tumor*”[tiab] OR “cancer*”[tiab] OR “Carcinoma*” [mh] OR “Neoplasm*”[mh] OR “solid tumors*”[tiab]) NOT (“review*” OR “systematic Review*”) | 1,832,989 |
| #2  Epidermal Growth Factor Receptor | (“Epidermal Growth Factor Receptor”[tiab] OR “EGFR” [tiab] OR “EGFR-targeted agents” [tiab]) NOT (“review*” OR “systematic Review*”) | 69,527 |
| #3  Near-infrared photoimmunotherapy | (“near-infrared photoimmunotherapy*”[tiab] OR “NIR-PIT”[tiab] OR “photoimmunotherapy*”[tiab] OR “RM-1929”[tiab] OR “ASP-1929”[tiab] OR “cetuximab sarotalocan”[tiab] OR “IRDye700DX/IR700” OR “cetuximab sarotalocan sodium”[tiab]) NOT (“review*” OR “systematic Review*”) | 559 |
|  | #1 AND #2 AND #3 | 72 |

Cochrane

| Concepts | Search string | Results (2015 – 2026) in trials |
| --- | --- | --- |
| #1  Tumour | Tumor AND cancer AND Carcinoma AND Neoplasm AND solid tumors | 153 |
| #2  Epidermal Growth Factor Receptor | Epidermal Growth Factor Receptor AND EGFR | 1256 |
| #3  Near-infrared photoimmunotherapy | near infrared photoimmunotherapy | 2696 |
|  | #2 AND #3 | 10 |
|  | #1 AND #3 | 9 |
|  | #1 AND #2 AND #3 | 9 |

Clinical Trials.gov

| Concepts | Search string | Results |
| --- | --- | --- |
| #1  Tumour | Tumor AND cancer AND Carcinoma AND Neoplasm AND Solid Tumors | 6391 |
| #2  Epidermal Growth Factor Receptor | Epidermal Growth Factor Receptor AND EGFR | 2232 |
| #3  Near-infrared photoimmunotherapy | “near-infrared photoimmunotherapy*” OR “NIR-PIT” OR “photoimmunotherapy*”OR “RM-1929”OR “ASP-1929”OR “cetuximab sarotalocan” OR “IRDye700DX/IR700” OR “cetuximab sarotalocan sodium” | 10 |
| #1 AND #2 AND #3 | Tumor AND cancer AND Carcinoma AND Neoplasm AND Solid Tumors AND Epidermal Growth Factor Receptor AND EGFR AND “near-infrared photoimmunotherapy*” OR “NIR-PIT” OR “photoimmunotherapy*”OR “RM-1929”OR “ASP-1929”OR “cetuximab sarotalocan” OR “IRDye700DX/IR700” OR “cetuximab sarotalocan sodium” | 10 |

Scopus

| Concepts | Search string | Results (2015 – 2026) |
| --- | --- | --- |
| #1  Tumour | TITLE-ABS-KEY ( Tumor OR cancer OR Carcinoma OR Neoplasm AND Solid Tumors ) AND PUBYEAR > 2014 AND PUBYEAR < 2027 AND ( EXCLUDE ( DOCTYPE,"re" ) ) AND ( LIMIT-TO ( LANGUAGE,"English" ) ) | 73,399 |
| #2  Epidermal Growth Factor Receptor | TITLE-ABS-KEY ( Epidermal Growth Factor Receptor* " AND " EGFR ) AND PUBYEAR > 2014 AND PUBYEAR < 2027 AND ( EXCLUDE ( DOCTYPE , "re" ) ) AND ( LIMIT-TO ( LANGUAGE , "English" ) ) | 34,026 |
| #3  Near-infrared photoimmunotherapy | TITLE-ABS-KEY ( "near-infrared photoimmunotherapy*" OR "NIR-PIT" OR "photoimmunotherapy*" OR "RM-1929" OR "ASP-1929" ) AND ( EXCLUDE ( DOCTYPE , "re" ) ) AND ( LIMIT-TO ( LANGUAGE , "English" ) ) | 962 |
| #1 AND #2 AND #3 | TITLE-ABS-KEY ( ( Tumor OR cancer OR Carcinoma OR Neoplasm AND Solid Tumors ) ) AND ( "Epidermal Growth Factor Receptor*" AND "EGFR" ) AND ( "near-infrared photoimmunotherapy*" OR "NIR-PIT" OR "photoimmunotherapy*" OR "RM-1929" OR "ASP-1929" ) AND ( EXCLUDE ( DOCTYPE , "re" ) ) AND ( LIMIT-TO ( LANGUAGE , "English" ) ) | 25 |

Embase

| Concepts | Search string | Results (2015 – 2026) |
| --- | --- | --- |
| #1  Tumour | ((tumor or cancer or carcinoma or neoplasm) and solid tumors). | 784 |
| #2  Epidermal Growth Factor Receptor | Epidermal Growth Factor Receptor and EGFR). | 535 |
| #3  Near-infrared photoimmunotherapy | near-infrared photoimmunotherapy/ or photoimmunotherapy/ or NIR-PIT/ or RM-1929/ or ASP-1929/ | 942 |
| #1 AND #2 AND #3 | (((tumor or cancer or carcinoma or neoplasm) and solid tumors and Epidermal Growth Factor Receptor and EGFR).mp. and near-infrared photoimmunotherapy/) or photoimmunotherapy/ or NIR-PIT/ or RM-1929/ or ASP-1929/ | 795 |

**Appendix 2: showing ROBINS-I checklist for Included Non-randomised Clinical trials and Interventional Studies.**

| Domains | Tahara et al., 2021[6] | Cognetti et al.,2025 [7] | Kadotal et al., 2025 [8] | Cognetti et al.,2021 [9] |
| --- | --- | --- | --- | --- |
| RoB from Confounding | 1.1 PN | 1.1 PY | 1.1 PN | 1.1 PN |
|  | 1.4 Y | 1.2 N | 1.4 Y | 1.4 Y |
|  | 1.5 Y | 1.3 PY | 1.5 Y | 1.5 Y |
|  | 1.6 PN | 1.7 Y | 1.6 PN | 1.6 PN |
|  |  | 1.8 Y |  |  |
| RoB Judgement |  |  |  |  |
|  | Low | Moderate | Low | Low |
| RoB from Participant selection | 2.1 N | 2.1 N | 2.1 N | 2.1 N |
|  | 2.4 Y | 2.4 Y | 2.4 Y | 2.4 Y |
| RoB Judgement |  |  |  |  |
|  | Low | Low | Low | Low |
| RoB from intervention classification | 3.1 Y | 3.1 Y | 3.1 Y | 3.1 Y |
|  | 3.2 Y | 3.2 Y | 3.2 Y | 3.2 Y |
|  | 3.3 N | 3.3 PN | 3.3 N | 3.3 PN |
| RoB Judgement |  |  |  |  |
|  | Low | Low | Low | Low |
| RoB from post-exposure intervention | 4.1 N | 4.1 N | 4.1 N | 4.1 N |
|  | 4.3 Y | 4.3 Y | 4.3 Y | 4.3 Y |
|  | 4.4 Y | 4.4 Y | 4.4 Y | 4.4 Y |
|  | 4.5 Y | 4.5 Y | 4.5 Y | 4.5 Y |
| RoB Judgement |  |  |  |  |
|  | Low | Low | Low | Low |
| RoB from missing data | 5.1 Y | 5.1 Y | 5.1 Y | 5.1 Y |
|  | 5.2 N | 5.2 N | 5.2 N | 5.2 N |
|  | 5.3 N | 5.3 N | 5.3 N | 5.3 N |
| RoB Judgement |  |  |  |  |
|  | Low | Low | Low | Low |
| RoB from Outcome measurement | 6.1 N | 6.1 N | 6.1 N | 6.1 N |
|  | 6.2 Y | 6.2 Y | 6.2 Y | 6.2 Y |
|  | 6.3 Y | 6.3 Y | 6.3 Y | 6.3 Y |
|  | 6.4 N | 6.4 N | 6.4 N | 6.4 N |
| RoB Judgement |  |  |  |  |
|  | Moderate | Moderate | Moderate | Moderate |
| RoB from report selection | 7.1 N | 7.1 N | 7.1 N | 7.1 N |
|  | 7.2 N | 7.2 N | 7.2 N | 7.2 N |
|  | 7.3 PN | 7.3 Y | 7.3 PN | 7.3 Y |
| RoB Judgement |  |  |  |  |
|  | Low | Moderate | Low | Moderate |
| Overall Bias Judgement | Low | Moderate | Low | Moderate |

Key: RoB- Risk of bias. Y- Yes. PY- Probably Yes. PN- Probably No. N-No. NI- No Information

Appendix 3: showing CASP Cohort checklist for Included Observational Studies (QoL, Prospective and Retrospective).

| CASP criteria for study review | | Okamoto et al., 2022b [18] | Ueda et al., 2025 [36] | Okamoto et al., 2025 [39] |  |
| --- | --- | --- | --- | --- | --- |
|  | Section A: Are the results of the review valid | | | | |
| Did the study address a clearly focused issue? | | YES | YES | YES |  |
| Was the cohort recruited in an acceptable way? | | YES | YES | YES |  |
| Was the exposure accurately measured to minimise bias? | | YES | YES | YES |  |
| Was the outcome accurately measured to minimise bias? | | YES | YES | YES |  |
| Have the authors identified all important confounding factors? | | Can't Tell | Can't Tell | YES |  |
| Have they taken account of the confounding factors in the design and/or analysis? | | Can't Tell | Can't Tell | YES |  |
| Was the follow up of subjects complete enough? | | YES | YES | YES |  |
| Was the follow up of subjects long enough? | | YES | YES | YES |  |
|  |  | | | | |
| What are the results of this study? | | YES | YES | YES |  |
| How precise are the results? | | YES | Can't Tell | YES |  |
| Do you believe the results? | | YES | YES | YES |  |
|  |  | | | | |
| Can the results be applied to the local population? | | YES | Can't Tell | YES |  |
| Do the results of this study fit with other available evidence? | | YES | YES | YES |  |
| What are the implications of this study for practice? | | Can't Tell | YES | YES |  |

Appendix 4: showing JBI Case Report checklist for Included Case Report Studies.

|  | Okamoto et al., 2022a [17] | Okamoto et al., 2022c [19] | Nishikawa et al., 2022 [20] | Omura et al., 2023 [23] | Okamoto et al., 2024 [28] | Kushihashi et al., 2024 [29] | Tamagawa et al.,2024 [30] | Nishimura et al., 2024 [31] | Kado et al., 2025 [33] | Takuma et al., 2025 [34] |  | Suekata et al., 2025 [35] |
| --- | --- | --- | --- | --- | --- | --- | --- | --- | --- | --- | --- | --- |
| Were patient’s demographic characteristics clearly  described? | Yes | Yes | Yes | Yes | Yes | Yes | Yes | Yes | Yes | Yes |  | Yes |
| Was the patient’s history clearly described and presented  as a timeline? | Yes | Yes | Yes | Yes | Yes | Yes | Yes | Yes | Yes | Yes |  | Yes |
| Was the current clinical condition of the patient on  presentation clearly described? | Yes | Yes | Yes | Yes | Yes | Yes | Yes | Yes | Yes | Yes |  | Yes |
| Were diagnostic tests or assessment methods and the  results clearly described? | Yes | Yes | Yes | Yes | Yes | Yes | Yes | Yes | No | Yes |  | Yes |
| Was the intervention(s) or treatment procedure(s) clearly  described? | Yes | Yes | Yes | Yes | Yes | Yes | Yes | Yes | Yes | Yes |  | Yes |
| Was the post-intervention clinical condition clearly  described? | Yes | Yes | Yes | Yes | Yes | Yes | Yes | Yes | Yes | Yes |  | Yes |
| Were adverse events (harms) or unanticipated events  identified and described? | Yes | No | Yes | Yes | Yes | Yes | Yes | Yes | Yes | Yes |  | Yes |
| Does the case report provide takeaway lessons? | Yes | Yes | Yes | Yes | Yes | Yes | Yes | Yes | Yes | Yes |  | Yes |

Appendix 5: showing JBI Case Series checklist for Included Case Series Studies.

|  | Shibutani et al., 2023 [25] | Makino et al., 2025 [32] |
| --- | --- | --- |
| Were there clear criteria for inclusion in the case  series? | Yes | Yes |
| Was the condition measured in a standard, reliable  way for all participants included in the case series? | Yes | Yes |
| Were valid methods used for identification of the  condition for all participants included in the case  series? | Yes | Yes |
| Did the case series have consecutive inclusion of  participants? | Yes | Yes |
| Did the case series have complete inclusion of  participants? | Yes | Yes |
| Was there clear reporting of the demographics of the  participants in the study? | Yes | Yes |
| Was there clear reporting of clinical information of the  participants? | Yes | Yes |
| Were the outcomes or follow up results of cases  clearly reported? | Yes | Yes |
| Was there clear reporting of the presenting  site(s)/clinic(s) demographic information? | Yes | Yes |
| Was statistical analysis appropriate? | Yes | Unclear |

Appendix 6: showing SYRCLE checklist for Pre-Clinical Animal Studies.

|  | Kato et al.,2023 [21] | Inagaki et al., 2023 [22] | Takao et al., 2023 [24] |  | Nagaya et al., 2015 [26] | Nagaya et al., 2018 [27] | Tanaka et al., 2026 [37] | Furumoto et al., 2022 [38] | Okada et al., 2022 [40] |
| --- | --- | --- | --- | --- | --- | --- | --- | --- | --- |
| Sequence Generation (Selection Bias) | Yes | Yes | Yes |  | Yes | Yes | Yes | Yes | Yes |
| Baseline Characteristics (Selection Bias) | Yes | Yes | Yes |  | Yes | Yes | Yes | Yes | Yes |
| Allocation concealment (Selection Bias) | Unclear | No | Unclear |  | Unclear | Unclear | Unclear | No | Unclear |
| Random housing (Performance bias) | Yes | Unclear | Yes |  | Yes | Yes | Yes | Yes | Yes |
| Blinding (Performance bias) | No | No | No |  | No | No | No | No | No |
| Random Outcome assessment (Detection bias) | No | No | No |  | No | No | No | No | No |
| Blinding (Detection bias) | No | No | No |  | No | No | No | No | No |
| Incomplete outcome data (Attrition bias) | Yes | Yes | Yes |  | Yes | Yes | Yes | Yes | Yes |
| Selective reporting outcome (Reporting bias) | Yes | Yes | Yes |  | Yes | Yes | Yes | Yes | Yes |
| Other sources of bias (Other) | Yes | Yes | Yes |  | Yes | Yes | Yes | Yes | Yes |
